# Parenteral glutamine supplementation in critical illness: a systematic review

**DOI:** 10.1186/cc13836

**Published:** 2014-04-18

**Authors:** Paul E Wischmeyer, Rupinder Dhaliwal, Michele McCall, Thomas R Ziegler, Daren K Heyland

**Affiliations:** 1 Department of Anesthesiology, University of Colorado School of Medicine, 12700 East 19th Avenue, Aurora, CO 80045, USA; 2 Clinical Evaluation Research Unit, Kingston General Hospital, 76 Stuart Street, Kingston, ON K7L 2V7, Canada; 3 Medical/Surgical Intensive Care Unit, St. Michael’s Hospital, 30 Bond Street, Toronto, ON M5B 1W8, Canada; 4 Department of Medicine, Center for Clinical and Molecular Nutrition, Emory University School of Medicine, 1648 Pierce Drive North East, Atlanta, GA 30322, USA; 5 Department of Medicine, Queens University, Kingston, Ontario, Canada

## Abstract

**Introduction:**

The potential benefit of parenteral glutamine (GLN) supplementation has been one of the most commonly studied nutritional interventions in the critical care setting. The aim of this systematic review was to incorporate recent trials of traditional parenteral GLN supplementation in critical illness with previously existing data.

**Methods:**

All randomized controlled trials of parenterally administered GLN in critically ill patients conducted from 1997 to 2013 were identified. Studies of enteral GLN only or combined enteral/parenteral GLN were excluded. Methodological quality of studies was scored and data was abstracted by independent reviewers.

**Results:**

A total of 26 studies involving 2,484 patients examining only parenteral GLN supplementation of nutrition support were identified in ICU patients. Parenteral GLN supplementation was associated with a trend towards a reduction of overall mortality (relative risk (RR) 0.88, 95% confidence interval (CI) 0.75, 1.03, *P* = 0.10) and a significant reduction in hospital mortality (RR 0.68, 95% CI 0.51, 0.90, *P* = 0.008). In addition, parenteral GLN was associated with a strong trend towards a reduction in infectious complications (RR 0.86, 95% CI 0.73, 1.02, *P* = 0.09) and ICU length of stay (LOS) (WMD –1.91, (95% CI -4.10, 0.28, *P* = 0.09) and significant reduction in hospital LOS (WMD -2.56, 95% CI -4.71, -0.42, *P* = 0.02). In the subset of studies examining patients receiving parenteral nutrition (PN), parenteral GLN supplementation was associated with a trend towards reduced overall mortality (RR 0.84, 95% CI 0.71, 1.01, *P* = 0.07).

**Conclusions:**

Parenteral GLN supplementation given in conjunction with nutrition support continues to be associated with a significant reduction in hospital mortality and hospital LOS. Parenteral GLN supplementation as a component of nutrition support should continue to be considered to improve outcomes in critically ill patients.

## Introduction

Glutamine (GLN) is the most abundant nonessential free amino acid [1] and has traditionally been classified as a nonessential amino acid able to be synthesized *de novo* in states of health. Glutamine is now commonly described as a conditionally essential amino acid, particularly in catabolic and stress states [2]. In catabolic states, large amounts of GLN are released from muscle tissue [3] as part of the body’s conserved evolutionary response to stress. Previous explanations for the release of GLN in periods of stress include use as a fuel source for rapidly dividing cells, a precursor for synthesis of nucleic acids, and a role in renal acid buffering [4,5]. Recent data has revealed that following illness and injury, GLN plays a vital role in inducing cellular protection pathways, modulation of the inflammatory response, and prevention of organ injury [1]. Contrary to long-held beliefs, not all critically ill patients become markedly deficient in the first few days of ICU admission, although some will present with severe depletion, such as is consistently seen in burn patients [6-8]. Recent data has shown GLN becomes depleted in approximately 25 to 35% of ICU patients at admission to ICU [6-8]. Patients with more prolonged critical illness, and in some studies, those receiving parenteral nutrition (PN) have shown show a greater level of depletion as described in a number of trials of PN-requiring ICU patients [9,10]. When GLN depletion is present at admission to ICU, it has been associated with increased mortality [7,8]. Clinical trials from the past 20 years demonstrated that GLN appears to reduce mortality, infectious complications, and ICU/hospital length of stay (LOS) [1]. Most of these previous trials were conducted in a select group of patients with the following characteristics: 1) predominantly receiving complete nutrition support via PN, 2) common exclusion of renal and liver failure based on glutamine product prescribing restrictions, 3) GLN supplementation of PN is often initiated later in the ICU stay (not at ICU admission during the shock phase), as PN is typically started later in the stay when enteral nutrition (EN) is not possible or meeting nutritional needs, 4) enrollment of oncology patients who may be at a greater risk for tumor-mediated GLN depletion, 5) use of GLN dose between 0.3 g/kg/day to a maximum of 0.5 g.kg/day given only via the intravenous route. This is summarized below:

Characteristics of traditional parenteral glutamine-supplementation trials

1. Enrolled patients requiring parenteral nutrition in most trials (85% of published trials)

2. Glutamine given as a supplement to nutrition support - not as a separate pharmaconutrient independent of nutrition support

3. GLN supplementation given with the start of PN later in the ICU stay, often when EN was not possible or failing to meet nutritional needs. (Not typically at ICU admission)

4. Common exclusion of renal and liver failure based on glutamine product prescribing restrictions (See Table 1 for specific trial exclusion criteria)

5. Lower GLN doses (0.3 to 0.5 g/kg/day)

6. Combined enteral and parenteral GLN not given

**Table 1:** Randomized studies evaluating glutamine (PN) in critically ill patients

**Study**	**Population**	**Exclusions for renal/liver failure**	**Methods (score)**	**Intervention dose of glutamine gm/kg/day**	**Mortality # (%)†**		**Infections # (%)‡**		**Length of stay (days)**	
					**Experimental**	**Control**	**Experimental**	**Control**	**Experimental**	**Control**
1) Griffiths 1997 & 2002 [28,29]	Single-center, mixed ICU, patients, N = 84	Not defined	C.Random: yes, ITT: yes, Blinding: yes (11)	PN, 0.26 IV glutamine + PN vs. PN, isocaloric, isonitrogenous	Hospital 18/42(43)	Hospital 25/42(60)	28/42 (67)	26/42 (62)	ICU 10.5 (6-19)*	ICU 10.5 (6-24)*
2) Powell-Tuck 1999 [36]	Single-center, mixed ICU/hospital, patients N = 168	Renal: creatinine >200 μmol/l Liver: if failure resulted in a PTR >1.8 or hepatic encephalopathy	C.Random: yes ITT: yes Blinding: yes (8)	PN, 0.26 IV glutamine + PN vs. PN, isocaloric, isonitrogenous.	Hospital 14/83(17)	Hospital 20/85(24)	NR	NR	Hospital 43.4 ± 34.1 (83)	Hospital 48.9 ± 38.4 (85)
3) Wischmeyer 2001 [40]	Single-center, critically ill burns N = 31	Renal: severe failure, Liver: severe failure	Random: not sure ITT: no Blinding yes (8)	PN, 0.57 IV glutamine + EN or EN + PN vs. AAcids + PN or EN or EN + PN, isocaloric, Isonitrogenous	Hospital 1/12 (8)	Hospital 4/14 (29)	7/12 (58)	9/14 (64)	Hospital 40 ± 10 (12)	Hospital 40 ± 9 (14)
4) Fuentes-Orozco 2004 [25]	Single-center, secondary peritonitis requiring TPN N = 33	Renal: creatinine >180 μmol/l. Liver: bilirubin >40 μmol/l, alanine aminotransferase >100 U/l and aspartate aminotransferase >100 U/l	C.Random: yes, ITT: yes. Blinding: double (11)	PN, 0.27 IV glutamine + PN vs. PN, isocaloric, isonitrogenous	Hospital 2/17 (12)	Hospital 3/16 (19)	4/17 (23)	12/16 (75)	ICU 7.2 ± 9.2 (17), Hospital, 16.5 ± 8.9 (17)	ICU 7.3 ± 4.5 (16) Hospital 16.7 ± 7 (16)
5) Zhou 2004 [42]	Severe burns N = 30	Renal: chronic disease. Liver: chronic disease	C.Random: yes. ITT: yes. Blinding: double (11)	PN, 0.35 IV glutamine + PN vs. PN, isocaloric, isonitrogenous.	NR	NR	3/15 (20)	4/15 (26)	Hospital 42 ± 7.0 (15)	Hospital 46 ± 6.6 (15)
6) Xian-Li 2004 [30]	Single-center, severe acute pancreatitis, N = 69	Renal: renal dysfunction. Liver: liver dysfunction	C.Random: yes. ITT: no Blinding: no (5)	PN, 0.4 IV glutamine + PN vs. PN	Hospital 0/20 (0)	Hospital 3/21 (14)	# Compl 4	# Compl 11	Hospital 25.3 ± 7.6 (20)	Hospital 28.6 ± 6.9 (21)
7) Dechelotte 2006 [9]	Multiple trauma, surgery, sepsis, pancreatitis from 16 ICUs N = 114									
	Renal: creatinine >250 μmol/L. Liver: Prothrombin time >23.7 sec	C. Random: NR. ITT: yes. Blinding: double (NA)	PN, 0.35 IV glutamine + PN vs. PN, isocaloric, isonitrogenous.	Hospital 2/58 (3), 6-month 16/58 (28)	Hospital 2/56 (3) 6-month 9/56 (16)	All 23/58 (40) Pneumonia 10/58 (17)	All 32/56 (58), Pneumonia 19/56 (34)	ICU 12.5 (1–430), Hospital 30 (1-560)	ICU 11.5 (3–121),* Hospital 26 (4-407)*	
8) Palmese 2006 [33]	Single-center, mixed ICU N = 84	Renal: severe renal disease. Liver: severe hepatic disease	C.Random: yes, ITT: yes, Blinding: single (10)	PN, 0.14 IV glutamine + EN with FOS vs. EN without FOS	ICU 6/42 (14)	ICU 8/42 (19)	All 13/42 (31), Pneumonia 2/42 (5)	All 21/42 (50), Pneumonia 6/42 (14)	ICU 12 ± 4.6 (42)	ICU 13 ± 3.4 (42)
9) Tian 2006 [38]	Single-center, MODS N = 40	Not defined	C.Random: not sure, ITT: yes, Blinding: none (6)	PN, 0.27 IV glutamine vs. PN	Unspecified 2/20 (10)	Unspecified 5/20 (25)	NR	NR	NR	NR
10) Sahin 2007 [37]	Single-center, acute pancreatitis N = 40	Not defined	C.Random: not sure, ITT: yes, Blinding: single (9)	PN, 0.3 IV glutamine + PN vs. PN, isocaloric, isonitorgenous.	Hospital 2/20 (10)	Hospital 6/20 (30)	NR	NR	Hospital 14.2 ± 4.4 (20)	Hospital 16.4 ± 3.9 (20)
11) Zhang 2007 [43]	Emergency and neurosurgical ICU, pts requiring PN for >7 days, N = 44	Not defined	C.Random: not sure, ITT: yes, Blinding: no (6)	EN and PN, IV glutamine 0.4 g/kg/day vs. EN and PN alone	NR	NR	NR	NR	ICU 11.73 ± 6.57 (22)	ICU 13.39 ± 5.08 (22)
12) Cai 2008 [21]	Single-center, elderly, severe sepsis N = 110	Renal: creatinine >220 μmol/L and/or dialysis. Liver: bilirubin >43 μmol/L and/or history of chronic liver disease	C.Random: not sure, ITT: yes, Blinding: no (10)	PN, 0.19 IV glutamine Patients received PN or EN + PN isocaloric, isonitrogenous	28-day 17/55 (31)	28-day 20/55 (36)	NR	NR	ICU 22.1 ± 4.9 (55)	ICU 23.8 ± 5.1 (55)
13) Duska 2008 [15]^∂^	Single-center, trauma N = 30	None	C.Random: not sure, ITT: yes, Blinding: single (8)	PN, 0.3 IV glutamine + PN vs. normal saline + supplemental PN, isocaloric, isonitrogenous	ICU 2/10 (20)	ICU 0/10 (0)	NR	NR	ICU 23 (median)	ICU 24 (median)
14) Estivariz 2008 [24]	Single-center, pancreatic and non pancreatic surgery N = 63	Renal: evolving acute renal failure or dialysis. Liver: total bili >4 mg/dL or >5 fold elevation in serum transaminase concentrations	C.Random: not sure, ITT: no**, Blinding: double (9)	PN, 0.5 IV glutamine + PN vs. PN isocaloric, isonitrogenous	Hospital 1/32 (3)	Hospital 6/31 (19)	Pneumonia 13/30 (43)	Pneumonia 16/29 (55)	ICU 12 ± 2 (32), Hospital 20 ± 2 (32)	ICU 23 ± 6 (31) Hospital 30 ± 6 (31)
17) Perez-Barcena 2008 [35]	Single-center, mixed ICU N = 30	Not defined	C.Random: not sure, ITT: yes, Blinding: single (10)	PN, 0.35 IV glutamine + PN vs. PN isocaloric, isonitrogenous	Hospital 3/15 (20)	Hospital 0/15 (0)	11/15 (73)	13/15 (87)	ICU 22.9 ± 20.6 (15), Hospital, 35.5 ± 33.6 (15)	ICU 20.5 ± 16.0 (15) Hospital 42.9 ± 28.8 (15)
18) Ozgultekin 2008 [32]	Single-center, CHI & GCS pts, ventilated, sedated, mean APACHE II 18-19 N = 60	Not defined	C.Random: not sure ITT: no, Blinding: none (4)	EN, 0.2-0.4 g/kg/d IV glutamine vs. EN	30-day 12/20 (60)	30-day 12/20 (60)	NR	NR	ICU 11.8 ± 5.9 (20)	ICU 17.3 ± 16.4 (20)
19) Yang 2008 [41]	Single-center, severe pancreatitis N = 61	Not defined	C.Random: not sure, ITT: no Blinding: single (4)	PN, IV glutamine (dose unknown) vs. PN, saline	Hospital 1/25 (4)	Hospital 3/25 (12)	NR	NR	Hospital 13.48 ± 1.42 (25)	Hospital 15.18 ± 1.14 (25)
20) Eroglu 2009 [23]	Single-center, severe trauma, ISS >20 N = 40	Renal: on renal replacement therapy, Liver: none	C.Random: yes, ITT: yes, Blinding: double (12)	EN, 0.5 g/kg/d IV glutamine vs. EN, saline	ICU 1/20 (5)	ICU 1/20 (5)	Overall 8/20 (40) VAP 1/20 (5)	Overall 10/20 (50), VAP 1/20 (5)	ICU 14 ± 2 (20)	ICU 15 ± 2 (20)
21) Perez-Barcena 2010 [34]	Single-center, trauma pt ISS >12, requires PN based on ASPEN N = 43	Not defined	C.Random: not sure, ITT: yes, Blinding: single (6)	PN, 0.35 g/kg/d IV glutamine vs. PN	ICU 4/23 (17), Hospital 0/23 (0)	ICU 2/20 (10), Hospital 1/20 (5)	Pneumonia 11/23 (48)	Pneumonia 8/20 (40)	ICU 21 (17–25),* Hospital 31 (19-42)*	ICU 21 (14-47)* Hospital 40 (24-80)*
22) Andrews 2011 [20]	Multi-center, critically ill adults, 25% medical pts, from 10 centres. N = 502	Renal: estimated GFR <10 ml/min and not receiving renal replacement therapy. Liver: none	C.Random: yes, ITT: yes, Blinding: double (13)	PN, 0.2-0.4 g/kg/day (20.2 g/day x 7 days) IV glutamine vs.PN isocaloric, isonitrogenous	ICU 88/250 (35), 6-month 115/250 (46)	ICU 80/252 (32), 6-month 106/252 (42)	134/250 (54)	131/252 (52)	ICU 15 (7.9-28.4)*, Hospital 32.5 (14.7-55.6)*	ICU 13.4 (8.2-23.9)*, Hospital 28.2 (15.1-52.4)*
23) Cekmen 2011 [22]	Single-center, mixed surgical ICU, ISS >10, APACHE II >10 N = 30	Renal: significant renal dysfunction (evolving acute renal failure or on dialysis). Liver: significant hepatic dysfunction (total bilirubin >4.0 mg/dL or more than fivefold elevation in serum transaminase concentrations)	C.Random: yes, ITT: yes, Blinding: double (10)	PN, 0.5 g/kg IV glutamine vs. PN	ICU (presumed) 3/15 (20)	ICU (presumed) 6/15 (40)	NR	NR	ICU 19.2 ± 12 (15)	ICU 27.4 ± 12 (15)
24) Grau 2011 [27]	Multi-center, mechanically ventilated, APACHE II >12, need TPN N = 127	Renal: chronic renal failure requiring dialysis, acute renal failure not treated with hemofiltration or hemodialysis [plasmatic creatinine >2.5 mg/dL]. Liver: hepatic failure with hepatic encephalopathy or portal hypertension	C.Random: not sure, ITT: yes Blinding: double (11)	PN, 0.5 g/kg IV glutamine vs. PN	ICU 9/59 (15), 6-month 16/59 (27)	ICU 13/68 (19), 6-month 23/68 (34)	All 24/59 (41), Surgical, 13/59 (22), Pneu (#/1000 vent days), 13.5, # infect/pt 1.5	All 31/68 (46), Surgical, 17/68 (25), Pneu (#/1000 vent days), 27.2, # infect/pt 2.4	ICU 12 (7-22)*, Hospital 35 (23-56)*	ICU 12 (7-24)*, Hospital, 31 (20-58)*
25) Wernerman 2011 [39]	Multi-center, mixed ICU, APACHE II >10 N = 413	Not defined	C.Random: yes, ITT: yes Blinding: double (11)	EN or PN, 0.28 g/kg/day IV glutamine vs. EN or PN, normal saline IV	ICU 8/205 (4) 28-day 14/205 (7)	ICU 11/208 (5) 28-day 20/208 (10)	NR	NR	NR	NR
26) Ziegler 2012 [44]	Multi-center, N = 150	Renal: history of chronic renal failure requiring dialysis, or significant renal dysfunction (creatinine >2.5 mg/dL and is not receiving continuous renal replacement therapy) or requiring acute hemodialysis postoperatively. Liver: current encephalopathy or known history of cirrhosis or total bilirubin ≥10.0 mg/dL	C.Random: yes ITT: yes, Blinding: double (12)	PN, 0.5 gm/kg/day IV vs. PN, isocaloric. Isonitrogenous.	Hospital, 11/75 (15)	Hospital 13/75 (17)	Any 33/75 (44). Pneumonia, 10/75 (13)			
	Any, 24/75 (32) Pneumonia, 12/75 (16)	ICU 17.5 ± 14.6 (75). Hospital 33.6 ± 28 (75)	ICU 13.6 ± 10 (75). Hospital 29.7 ± 20.7 (75)							

^†^Hospital mortality unless stated otherwise; ^‡^number of patients with infections unless stated otherwise; ^*^data for length of stay is in median and ranges; ^**^data for mortality is ITT, infections is non-ITT; ^∂^data from growth hormone group not shown here. ± ( ), mean ± standard deviation (number); Compl, complications; ICU, intensive care unit; Infec/pt, infections per patient; C.Random, concealed randomization median (range); Pneu, pneumonia; PN, parenteral nutrition; ITT, intention to treat; EN, enteral nutrition; TPN, total parenteral nutrition; NR, not reported; FOS, fructooligosaccharides; MODS, multiple organ dysfunction syndrome; CHI, closed head injury; GCS, Glasgow Coma Scale; APACHE II, Acute Physiology and Chronic Health Evaluation II; ISS, injury severity score; Vent days, ventilator days; VAP, ventilator-associated pneumonia; ASPEN, American Society for Parenteral and Enteral Nutrition; GFR, glomerular filtration rate; NA, not applicable.

Recently, a new paradigm of pharmacological GLN administration was tested in a large multicenter clinical trial utilizing a combined enteral and intravenous dose of GLN, which averaged between 0.6 to 0.8 g/kg/day independent of the administration of complete nutrition [6]. This study, the REDOXS study, also primarily enrolled patients who, as defined by the inclusion criteria, were required to be in multi-system organ failure within 24 hours of admission. Finally, renal failure and acute liver failure were, for the first time, not excluded and in fact, more than 30% of the patients in REDOXS presented with baseline acute renal failure. The results of this trial surprisingly showed contrary to many previous traditional PN-based GLN trials, that GLN supplementation was associated with an increase in mortality.

Thus, a key question to be answered is whether parenteral GLN administered as a supplement to complete nutrition support (for example via PN) is beneficial or harmful. This question is timely given the large number of (n = 11) of randomized clinical trials of GLN supplementation of PN published since 2009. Recently a number of meta-analysis have been published examining the use of GLN in burn injury, pancreatitis, and surgical and critical illness combined [11-14], however a systematic analysis focused on parenteral GLN supplementation in critical illness has not been performed. Further, these past meta-analysis have not incorporated all of the most recently available trial data. The aim of this current systematic literature review and meta-analysis is to focus on the question of whether the ‘traditional’ parenteral administration of GLN as part of nutrition support has an effect on relevant clinical outcomes in patients classified with critical illness (as opposed to elective surgery or only single diagnosis groups, such as burn injury).

## Methods

### Study identification

The following databases were searched for articles from 1980 until July 2013: EMBASE, MEDLINE, CINAHL and the Cochrane Controlled Trials Register and Database of Systematic Reviews. The literature search used broad search terms containing ‘randomized’ , ‘blind’ , ‘clinical trial’ , ‘nutrition’ , ‘nutritional support’ or ‘dietary supplementation’ or ‘enteral nutrition’ or ‘parenteral nutrition’ or ‘parenteral nutrition solutions’ and ‘critical care’ or ‘critical illness’ or ‘intensive care units’. The results were then reviewed to identify articles using parenteral or intravenous GLN supplementation. A unique feature of this meta-analysis is that no language restrictions were placed on the searches. Personal files and reference lists of relevant review articles were also reviewed. As this was a systematic review no ethics board approval or patient consent was required.

### Study selection criteria

We only included original studies if they met the following inclusion criteria: a) study design: randomized clinical trials, b) population: critically ill adult patients (>18 years of age), defined as patients admitted to an ICU. When this was unclear, we considered a mortality rate higher than 5% (hospital mortality and if this was not reported we used ICU mortality or 28-day mortality) in the control group to be consistent with critical illness, c) intervention: parenteral GLN versus control (either isonitrogenous amino acid control) or placebo, d) study outcomes: must have included one of the following: mortality, ICU and hospital LOS, infectious complications, and other clinically important complications. Studies of enteral GLN only or combined enteral/parenteral GLN were excluded).

### Data abstraction

Decisions about the inclusion of the articles were made in duplicate. All original studies were reviewed independently by two reviewers using a data abstraction form with a scoring system as shown in Table 2. An assessment of the criteria for inclusion, details on the patient population, intervention and control/placebo and clinical outcomes were made as per earlier publications [15]. The methodological quality of individual trials was also assessed according to a) whether randomization was concealed, b) blinding, c) analysis was based on the intention-to-treat (ITT) principle, d) patient selection, e) comparability of groups at baseline, f) extent of follow-up, g) description of treatment protocol and co-interventions, and h) definition of clinical outcomes. Each individual study was scored from 1 to 14 (Table 2). Disagreement in the individual scores of each of the categories was resolved by consensus between both reviewers. We attempted to contact the authors of included studies and requested additional information not contained in published articles.

**Table 2:** Randomized trial quality scoring system

	**Score**		
	**0**	**1**	**2**
**Randomization**	…	Not concealed or not sure	Concealed randomization
**Analysis**	Other	…	Intention-to-treat
**Blinding**	Not blinded	Single-blind	Double-blinded
**Patient selection**	Selected patients or unable to tell	Consecutive eligible patients	…..
**Comparability of groups at baseline**	No or not sure	Yes	….
**Extent of follow-up**	<100%	100%	….
**Treatment protocol**	Poorly described	Reproducibly described	….
**Co-interventions**	Not described	Described but not equal or not sure	Well described and all equal
**Outcomes**	Not described	Partially described	Objectively defined

### Data synthesis

The primary outcome of the systematic review was overall mortality. From all studies, we combined hospital mortality where reported (specified or assumed to be hospital if not specified). If hospital mortality was not reported, we used ICU mortality or, if ICU mortality was not reported, we used 28-day mortality. Secondary outcomes included infection, ventilator-associated pneumonia (VAP), and ICU and hospital LOS. We used definitions of infections as defined by the authors in their original articles. We combined data from all trials to estimate the pooled risk ratio (RR) with 95% confidence intervals (CIs) for mortality, infectious complications, VAP and overall weighted mean difference (WMD) with 95% CIs for LOS data. Pooled RRs were calculated using the Mantel-Haenszel estimator and WMDs were estimated by the inverse variance approach. The random effects model of Der Simonian and Laird was used to estimate variances for the Mantel-Haenszel and inverse variance estimators [16]. All analyses, except the test for asymmetry, were conducted using Review Manager (RevMan) 5.1 software. (The Nordic Cochrane Centre, The Cochrane Collaboration, Copenhagen, Denmark, 2011) [17].

When possible, studies were aggregated on an ITT basis (Table 2). The presence of heterogeneity was tested by a weighted Mantel-Haenszel chi-square test and quantified by the I^2^ statistic as implemented in RevMan 5.1 [17,18]. The possibility of publication bias was assessed by generating funnel plots and testing asymmetry of outcomes using methods proposed by Rucker and colleagues [19]. We considered *P* <0.05 to be statistically significant and *P* <0.20 as the indicator of trend.

### Subgroup analyses

We utilized predefined subgroup analysis to assess a number of possible influences on the effect of parenteral GLN supplementation on clinical outcomes. We first examined the effect of parenteral GLN supplementation of PN versus EN alone. As trials examining EN alone consistently deliver significantly less total overall energy and protein delivery, we hypothesized that parenteral GLN may have a different effect when complete nutrition support was being given via EN alone or as a component of PN delivery. We next assessed the effect of trial quality on outcome as it is often hypothesized trials of lower methodological quality tend to yield more positive clinical signals for the therapy being tested when compared to trials of higher methodological quality. Utilizing our trial scoring tool, we designated trials with a methodological score of >8 as a ‘high-quality trial’ for the purposes of this review. Finally, it has been postulated that single-center trials may yield a greater chance of a positive clinical signal versus more rigorous multi-center trials [16], thus we examined the effect of these two types of trials on our clinical outcomes.

## Results

### Study identification and selection

The literature search yielded 58 potentially eligible randomized controlled trials of which 26 were included in our systematic review. As shown in Table S1 in Additional file 1, a total of 32 studies were excluded for these reasons: 1) patients not considered to be critically ill (n = 18), 2) no clinical outcomes (n = 5), 3) were duplicate studies or subgroups of included studies (n = 3), 4) crossover design studies (n = 2), 5) varying doses of GLN (n = 1), 6) a trial of combined enteral and parenteral GLN (n = 1), 7) questionably low dosage of GLN (0.002 g/kg/day) (n = 1, see Table S1 in Additional file 1) and 8) one trial reported data from a subgroup of its total study population [10], see Table S1 in Additional file 1).

Thus, we included 26 studies of parenteral GLN supplementation performed in ICU patients with diagnosis ranging from pancreatitis, trauma, burns to sepsis described in Table 1[9,15,20-44]. While in the majority of the studies, the intervention and control groups received GLN-free parenteral nutrition/amino acids, in four studies, patients received only EN as the sole source of nutrition support [23,31-33].

### Meta-analyses of primary and secondary outcomes

#### Effect of GLN supplementation on mortality

**Figure 1:**
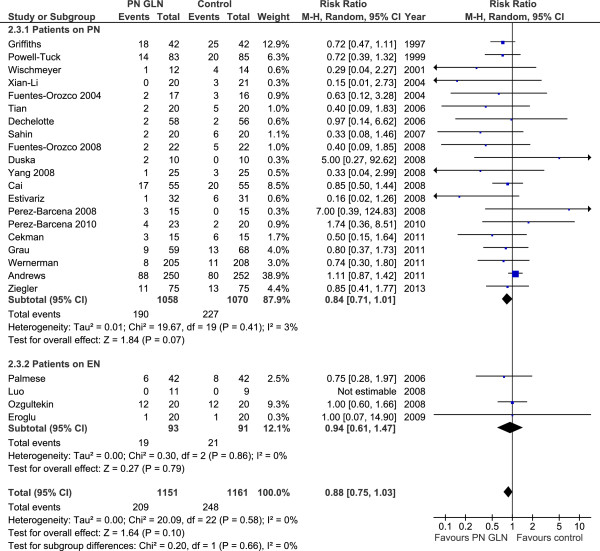
Overall mortality.

**Figure 2:**
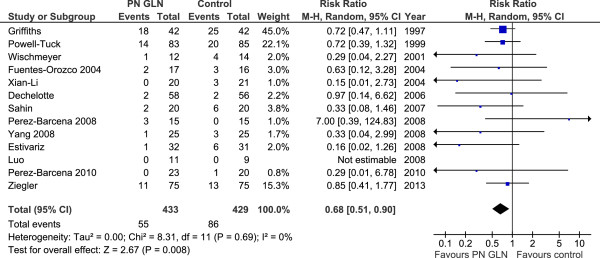
Hospital mortality.

When the 24 studies that reported on mortality (Figure 1) were aggregated, IV GLN supplementation was associated with a trend towards a reduction in overall mortality (RR 0.88, 95% CI 0.75, 1.03, *P* = 0.10, heterogeneity I^2^ = 0%). In the 13 studies (Figure 2) that reported hospital mortality, a significant reduction in hospital mortality was seen when they were aggregated (RR 0.68, 95% CI 0.51, 0.90, *P* = 0.008, heterogeneity I^2^ = 0%).

#### Effect of GLN supplementation on infectious complications, ICU length of stay and hospital length of stay

**Figure 3:**
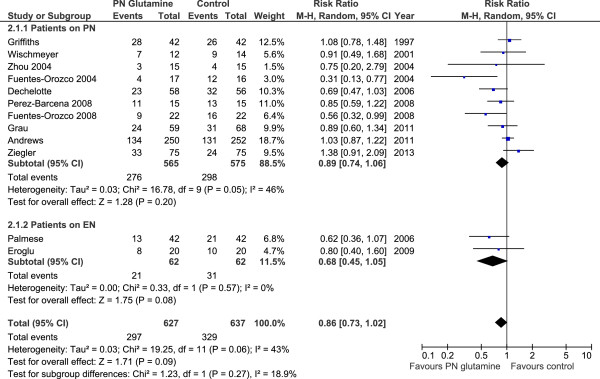
Infectious complications.

**Figure 4:**
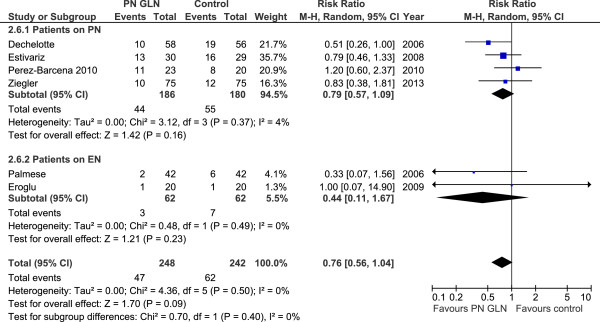
Ventilator-associated pneumonia.

When the 12 studies (Figure 3), which reported infectious complications, were aggregated, GLN supplementation was associated with a trend towards a reduction in infectious complications (RR 0.86, 95% CI 0.73, 1.02, *P* = 0.09, heterogeneity I^2^ = 43%). When the six studies that reported VAP (Figure 4) were aggregated, GLN supplementation was associated with a trend towards a reduction in pneumonia (RR 0.76, 95% CI 0.56, 1.04, *P* = 0.09, heterogeneity I^2^ = 0%).

**Figure 5:**
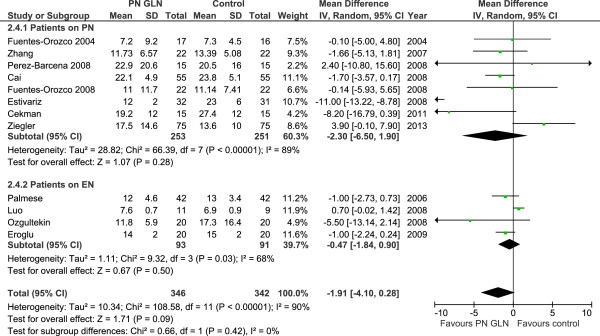
ICU length of stay.

**Figure 6:**
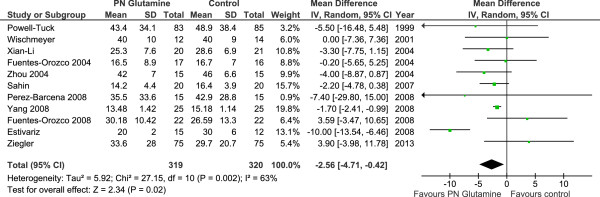
Hospital length of stay.

When the 11 studies that reported ICU length of stay as a mean ± standard deviation were aggregated (Figure 5), GLN supplementation was associated with a trend to reduction in ICU LOS (WMD -1.91, 95% CI -4.10, -0.28, *P* = 0.09, heterogeneity I^2^ = 90%). Finally, when the 11 studies reporting on hospital LOS were aggregated (Figure 6), GLN supplementation was associated with a significant reduction in hospital LOS (WMD -2.56, 95% CI -4.71, -0.42, *P* = 0.02, heterogeneity I^2^ = 63%).

### Subgroup analysis

Results across all these subgroups are reported in Table 3. Overall tests for significance between subgroups revealed no statistically significant differences between any individual subgroups.

**Table 3:** Subgroup analyses

	**Overall mortality**				**Hospital mortality**				**Infectious complications**				**ICU LOS**				**VAP**			
**Subgroup analysis**	**Number of trials**	**Number of patients**	**Effect on overall mortality [RR (95% CI), *p*]**	**Test for subgroup differences**	**Number of trials**	**Number of patients**	**Effect on hospital mortality [RR (95% CI), *p*]**	**Test for subgroup differences**	**Number of trials**	**Number of patients**	**Effect on infectious complications [RR (95% CI), *P*]**	**Test for subgroup differences**	**Number of trials**	**Number of patients**	**Effect on ICU LOS [WMD (95% CI), *P*]**	**Test for subgroup differences**	**Number of trials**	**Number of patients**	**Effect on VAP [RR (95% CI), *P*]**	**Test for subgroup differences**
*Study Quality*																				
Low quality (<8)	5	214	0.81 (0.44, 1.48), *P* = 0.49	*P* = 0.79	3	134	0.26 (0.06, 1.19), *P* = 0.08	*P* = 0.21	NA				NA				NA			
High quality (≥8)	19	2103	0.88 (0.74, 1.04) *P* = 0.12		10	733	0.70 (0.52, 0.94), *P* = 0.02													
*Number of sites*																				
Single center	19	1011	0.75 (0.60, 0.93), *P* = 0.009	*P* = 0.04	11	603	0.64 (0.47, 0.88), *P* = 0.006	*P* = 0.45	8	371	0.77 (0.60, 0.98), *P* = 0.03	*P* = 0.16	NA				4	226	0.86 (0.58, 1.28), *P* = 0.45	*P* = 0.34
Multi-center	5	1306	1.03 (0.83,1.28) *P* = 0.79		2	264	0.86 (0.43, 1.71), *P* = 0.67		4	893	0.97 (0.77, 1.23) *P* = 0.83						2	264	0.63 (0.38, 1.04) *P =* 0.07	
*PN vs. EN*																				
Patients on PN	20	2128	0.84 (0.71, 1.01) *P =* 0.07	*P* = 0.66	NA				10	1140	0.89 (0.74, 1.06) *P = 0.20*	*P* = 0.27	8	354	-2.30 (-6.50, 1.90) *P* = 0.28	*P* = 0.42	4	366	0.79 (0.57, 1.09) *P =* 0.16	*P* = 0.40
Patients on EN	4	184	0.94 (0.61, 1.47), *P* = 0.79						2	124	0.68 (0.45, 1.05), *P* = 0.08		3	184	-0.47 (-1.84, 0.90) *P =* 0.50		2	124	0.44 (0.11, 1.67), *P* = 0.23	

ICU, intensive care unit; LOS, length of stay; VAP, ventilator-associated pneumonia; RR, relative risk; WMD, weighted mean difference; CI, confidence interval; *P*, *P* value; EN, enteral nutrition; PN, parenteral nutrition; NA, not applicable.

#### Effect of parenteral GLN supplementation of PN versus EN alone

Examining the effect of GLN supplementation of PN versus EN alone, the test for subgroup differences in overall mortality outcomes was not significant (*P* = 0.66). In the studies in which patients received intravenous (IV) GLN plus PN, GLN supplementation was associated with a trend towards a reduction in overall mortality (RR 0.84, 95% CI 0.71, 1.01, *P* = 0.07, heterogeneity I^2^ = 3%; Figure 1, Table 3). Only four studies reported on mortality for GLN supplementation of EN. When the studies in which patients only received IV GLN and EN [23,31-33] were aggregated, GLN supplementation had no effect on overall mortality (RR 0.94, 95% CI 0.61, 1.47, *P* = 0.79, heterogeneity I^2^ = 0%; Figure 1, Table 3). In the subgroup of studies in which patients received IV GLN plus PN, GLN supplementation had no effect on infectious complications (RR 0.89, 95% CI 0.74, 1.06, *P* = 0.20, heterogeneity I^2^ = 48%; Figure 3, Table 3). However, for the subgroup of studies in which patients received IV GLN and were on EN alone [23,33], GLN supplementation was associated with a trend towards a reduction in infectious complications (RR 0.68, 95% CI 0.45, 1.05, *P* = 0.08, heterogeneity I^2^ = 0%; Figure 3, Table 3). The test for subgroup differences in infectious morbidity overall in EN alone versus PN studies was not significant (*P* = 0.27).

For the subgroup of studies in which patients received IV GLN plus PN, GLN supplementation was not associated with a reduction in ICU LOS. Similarly no effect on ICU LOS was observed in patients receiving only EN that received IV GLN [23,31-33]. The test for subgroup differences in ICU LOS overall was not significant (*P* = 0.42). None of the studies in which patients only received EN reported on hospital LOS and therefore no subgroup analyses were done.

#### Effect of study quality on outcomes

Higher quality trials (methodological score of >8) showed a stronger statistical trend towards reduced mortality than trials of lower quality (*P* = 0.12 versus *P* = 0.49 respectively), although the effect size was similar between the groups (Table 3). The overall test for subgroup difference was not significant for these subgroups (*P* = 0.79).

A statistically significant effect for GLN reducing hospital mortality was only seen in the higher quality trials, although there were a smaller number of patients enrolled in the lower quality trials and this reduced the statistical power of the signal (Table 3). Again, overall tests for significance did not reveal statistically significant differences between these subgroups (*P* = 0.21). There were insufficient numbers of trials reporting on infectious outcomes and LOS data in the low- and high-quality categories to allow for these comparisons to be made.

#### Role of multi-center versus single-center trials on outcomes

The role of multi-center versus single center trials revealed that only the single center trials demonstrated a significant effect of GLN on overall and hospital mortality and infectious outcomes (Table 3). Only the multi-center trials demonstrated a significant benefit of GLN on the incidence of VAP (Table 3). Overall tests for significance did not reveal statistically significant differences between these subgroups. (*P* = 0.45 for mortality and *P* = 0.16 for infectious morbidity, and *P* = 0.34 for VAP).

### Risk of bias across studies

**Figure 7:**
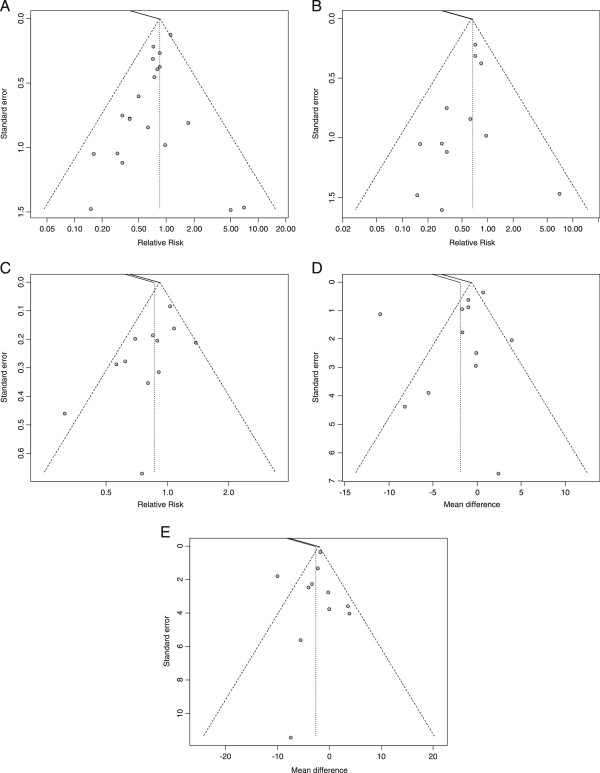
**Funnel plots of primary and secondary outcomes. (A)** Overall mortality: (Test for asymmetry p=0.57); **(B)** hospital mortality: (Test for asymmetry p=0.86); **(C)** infection: (Test for asymmetry p=0.05); **(D)** ICU LOS: (Test for asymmetry p=0.87); **(E)** hospital LOS: (Test for asymmetry p=0.69). LOS, length of stay.

Funnel plots for all outcomes were created to assess for publication bias as shown in Figure 7. The test of asymmetry was not found to be significant for any of the endpoints including overall mortality (*P* = 0.57), hospital mortality (*P* = 0.86), infectious complications (*P* = 0.05) ICU LOS (0.87), or hospital LOS (0.69).

## Discussion

Meta-analysis of the initial randomized controlled trials of parenteral GLN supplementation suggested outcome benefits in the six early trials examining critical illness [45]. Since that time an additional 22 randomized clinical trials have been performed in over 2,000 patients receiving GLN as a component of complete nutrition support, primarily given as PN (85% of trials). In addition, we have a much greater understanding of the potential beneficial mechanisms of GLN in critical illness as summarized in recent reviews [1,46].

Early trials of GLN were primarily focused on patients receiving full nutrition support primarily by PN at doses falling within the approved prescribing indications for dose in commercially available GLN preparations. Further, as shown in Table 1, patients in these traditional trials were commonly excluded from enrollment if they had pre-existing renal or liver failure. These traditional nutritionally oriented (or non-pharmaconutrition based) GLN supplementation trials have shown a consistent reduction of mortality and benefit on other outcomes as suggested by the 2009 update of the Canadian Critical Care Nutrition Guidelines [47]. However, given the publication of 11 new randomized controlled trials examining the traditional parenteral use of GLN as a component of nutrition support (predominantly PN) since 2009, a new systematic review is indicated.

This need for a new systematic review is further driven by the results of the REDOXS trial, a 1,200-patient, 40-center randomized controlled trial of ‘pharmacologically dosed’ parenteral and enteral GLN (approximately 0.6 to 0.8 g/kg/day) factorialized with antioxidant supplementation [6]. This trial was distinct from any of the previously published parenteral GLN trials supplementing PN or EN in ICU patients as GLN (and a cocktail of antioxidants) were administered independent of any concomitant nutrition support. In contrast to the design of studies reported in this analysis, the nutritional delivery of energy and protein in the REDOXS trial averaged less than 50% of that prescribed for the patient, and thus was quite insufficient in meeting patient needs. In addition, all patients in the REDOXS trial had documented multi-system organ failure at enrollment, which is a common exclusion criteria in the trials reported in this analysis using parenteral GLN as a supplement to nutrition support. Further, more than 30% of REDOXS patients were found to have baseline renal failure at admission, which was a very common exclusion criteria in the trials reported in the analysis (Table 1). As stated, this is a key difference of REDOXS from all other GLN trials.

Thus, given the many differences in fundamental trial design between REDOXS and traditional GLN supplementation trials, the REDOXS trial has not been included in this systematic review focused on GLN supplementation of nutrition support. However, the publication of the REDOXS trial necessitates a close examination of the many new GLN supplementation trials published recently to examine if there has been a change in the fundamental signal of benefit of GLN supplementation of PN seen in historical trials in the recent era of critical care practice. This was the primary aim of this systematic review.

Overall, our results reveal that GLN supplementation continues to show a significant reduction in hospital mortality. In addition, GLN supplementation of complete nutrition was found to be associated with a strong trend towards a reduction in infectious complications, ICU LOS and a significant reduction in hospital LOS. GLN supplementation as part of nutrition support also continues to show a trend towards reduction of overall mortality in all trials examining both PN and EN nutrition delivery. The signal may be stronger for trials that utilize GLN supplementation as part of PN nutrition delivery. Importantly, no statistically significant signal of publication bias was observed in any of our tests for asymmetry as shown in the funnel plots in Figure 7.

A number of high-quality multi-center trials of parenteral GLN supplementation have been published recently, driving the need for this new critical care focused systematic review. A number of these key trials have strengths and weaknesses that are in some cases not reflected in the trial quality scoring utilized by this and other systematic reviews. Thus, a brief discussion of some of these key trials is useful. In short, these trials include the Scandinavian GLN trial [39], which examined 413 patients in multiple centers and demonstrated a benefit on ICU survival rates with parenteral GLN. A limitation of this trial was that it was stopped prior to achievement of its predefined enrollment goal due to slow enrollment. Another multi-center trial, the SIGNET trial, showed no benefit of short-term (approximately ≤4 days), low-dose GLN (approximately 0.2 g/kg/d) given parenterally to ICU patients on parenteral feeding [20]. Some concerns with this trial included lack of availability of some key data including issues regarding missing values, dropouts, protocol violations, and complete follow-up data. The actual values for GLN doses based on body weight have not been published and this has been viewed as a significant limitation of the trial. In an attempt to address the effect of quality of the trials and single-center versus multi-center trials included in the systematic review, we performed subgroup analyses to examine the role of these factors. We observed a more significant signal for parenteral GLN supplementation reducing mortality in the higher quality studies versus the lower quality trials. Single-center trials showed a greater benefit of GLN supplementation on mortality and overall infectious morbidity, although no signals of harm were observed in the multi-center trials of GLN on mortality. Interestingly, the beneficial effect of GLN on VAP was greater in the multi-center trials then the single-center trials. This is an endpoint that may deserve more focused examination in future trials of GLN supplementation in the ICU.

A number of other systematic reviews of GLN supplementation in either much broader populations, including noncritically ill patients, or specific populations (pancreatitis, burn injury, or elective surgery) have been published recently [11-14]. To our knowledge, our systematic analysis is the first to focus on the parenteral use of GLN supplementation of standard nutrition support in which all subjects are critically ill. When compared to the largest recent meta-analysis in a broader mixed elective surgery and critical care population many similarities with our data are observed [11]. First, with regard to mortality this recent analysis of GLN showed a very similar trend towards reduction of mortality (RR = 0.89; 95% CI, 0.77 to 1.04 versus RR 0.88, 95% CI 0.75, 1.03, in our analysis) in a patient population with a lower illness severity (where an effect on mortality would be less likely to be observed). No analysis of hospital mortality was reported in this previous analysis. With regard to infectious morbidity, this earlier meta-analysis showed a stronger statistically significant reduction of infectious morbidity (RR = 0.83; 95% CI, 0.72 to 0.95) compared to our data where a nonsignificant trend of similar magnitude for a benefit on infection was observed (RR 0.86, 95% CI 0.73, 1.02). Both the previous and current systematic reviews reported significant reductions on hospital LOSs of approximately 2.5 days (-2.35; 95%, -3.68 to -1.02 versus -2.56, 95% CI -4.71, -0.42 in our analysis). Other key differences of our analysis versus the earlier report was that no direct industry funding was involved in support of our analysis (partial funding by an unrestricted industry grant was used to support this previous analysis), we included all of the world literature (including non-English trials) in our search, and we successfully attempted in all cases to contact the original authors when any questions of missing data or actual ICU patient inclusion was in question. For the first time we also examined the effect of GLN supplementation on VAP. We were also able to incorporate original source data from the unpublished multi-center American ‘GLND’ trial of GLN-supplementation of PN published in abstract form [44]. Finally, we performed funnel plots for all primary and key secondary endpoints examined by this analysis to examine possible publication bias associated with these endpoints.

The strength of our meta-analysis includes the use of several methods to reduce bias (comprehensive literature search, duplicate data abstraction, specific criteria for searching and analysis) and focus on clinically important primary outcomes. Notwithstanding, we are aware that our meta-analysis has several limitations. The major limitation is the small number of trials included in certain subgroup analyses such as PN-supplementation of EN. We also unfortunately could not perform subgroup analysis for all endpoints due to limited numbers of trials examining the particular endpoints. Another potential weakness of any systematic review of randomized controlled nutrition trials has been pointed out by Vincent *et al*. recently [48]. This is the potential inability of a controlled nutrition trial to recreate ‘real-life’ patient conditions in the controlled setting of a trial (that is due to many patient exclusions). That said, we feel this systematic review best reflects all existing data in a wide variety of critical care settings of parenteral GLN supplementation to provide clinicians with most complete data to assist in making clinical decisions. Further, despite our attempts to be fully comprehensive in our search for all available trials we may not have been to include all available trials.

In spite of these limitations, we have demonstrated that traditional GLN supplementation used in the context of standard (predominantly parenteral) nutrition support in the critically ill may significantly reduce hospital mortality and shorten hospital LOS with a trend towards reduction in overall mortality and infectious complications, including VAP, and ICU LOS. Nonetheless, many questions on the ideal dose and timing of GLN supplementation in the ICU still remain unanswered. Further research is warranted to define the optimal dose and timing of supplementation of GLN in patients receiving full nutrition support. Recent data from REDOXS and other trials suggest that parenteral GLN should not be given in patients early in the acute phase of critical illness, in patients with multiple organ failure or in patients with unresuscitated shock requiring significant vasopressor support. Finally, based on the results of the REDOXS trial [6], we believe that high-dose parenteral or parenteral + enteral GLN (doses >0.5 g/kg/d) should not be used during the acute phase of critical illness.

## Conclusions

In this comprehensive systematic review, we demonstrate that traditional parenteral GLN supplementation as a component of nutrition support (primarily added to PN) is associated with a significant decrease in hospital mortality and length of hospital stay. GLN supplementation is also associated with trends towards reduced overall mortality, infectious complications and ICU LOS in critically ill patients. The therapeutic effect may be dependent on GLN dose given, with optimal benefit traditionally observed between 0.3 and 0.5 g/kg/d. Thus, we recommend that parenteral GLN supplementation as a component of nutrition support be considered as an approach to improve outcomes of critical illness in selected patients. Our data here suggest that parenteral GLN supplementation, as a component of complete PN and/or EN support, is safe when administered following resolution of shock and multi-organ failure, and with daily doses less the 0.5 g/kg/d. Focused clinical trials on the clinical efficacy of parenteral GLN supplementation combined with adequate and complete specialized nutrition support are needed in critically ill patients at risk of GLN depletion who do not have multiple organ failure or ongoing shock.

## Key messages

• Critical illness is characterized by severe metabolic stress and glutamine depletion has been associated with increased mortality in some recent studies. In this context, supplementation of parenteral glutamine, predominantly as a component of parenteral nutrition, may improve clinical outcomes when given to appropriate patients as part of complete nutrition support.

• Supplemental parenteral glutamine may significantly decrease hospital mortality and shorten hospital LOS in critically ill patients.

• Supplemental parenteral glutamine given as a component of nutrition support is associated with a trend towards a reduction in overall mortality, infectious morbidity, and ICU LOS in critically ill patients.

• We suggest supplemental glutamine should not be given in a high dose (>0.5 g/kg/day) or in patients early in the acute phase of critical illness in patients with multiple organ failure or unresuscitated shock requiring significant vasopressor support.

• When parenteral nutrition is prescribed to critically ill patients, parenteral supplementation with glutamine should continue to be considered safe and may potentially improve outcomes in the ICU in patients without specific contraindications.

## Abbreviations

CI: confidence interval; EN: enteral nutrition; GLN: glutamine; ICU: intensive care unit; ITT: intention to treat; IV: intravenous; LOS: length of stay; PN: parenteral nutrition; RR: relative risk; VAP: ventilator-associated pneumonia; WMD: weighted mean difference.

## Competing interests

No funding for the development, writing, or submission of this manuscript was received. Paul Wischmeyer has research funding for the REDOXS trial of glutamine and antioxidants from Fresenius Kabi. Rupinder Dhaliwal has no conflict of interest to declare. Michele McCall has no conflict of interest to declare. Thomas R. Ziegler has research funding for the GLND trial of glutamine-supplemented total parenteral nutrition in surgical critical care from Fresenius Kabi. Daren K. Heyland has research funding for the REDOXS trial of glutamine and antioxidants from Fresenius Kabi.

## Authors’ contributions

PW contributed to development of the manuscript concept, study grading, study selection, evaluation, interpretation of data and performed the primary authoring and editing of the manuscript. RD contributed to development of the manuscript concept, study grading selection, evaluation, interpretation of data and performed much of the primary statistical analysis and meta-analysis data analysis. He also performed significant work authoring and editing all drafts of the manuscript. MM contributed to development of the manuscript concept, study grading, study selection, evaluation, interpretation of data and assisted in editing the manuscript. TZ assisted in editing the manuscript drafts and confirmed the primary data of the GLND trial. DH contributed to development of the manuscript concept, study grading, study selection, evaluation, interpretation of data and assisted in the primary editing of all drafts of the manuscript. All authors read and approved the final manuscript.

## Supplementary Material

Additional file 1: Table S1 Studies of glutamine supplementation in patients not included in the analysis. Click here for file

## References

[B1] Wischmeyer PE Glutamine: role in critical illness and ongoing clinical trials Curr Opin Gastroenterol 2008 24 190 197 10.1097/MOG.0b013e3282f4db94 18301270

[B2] Coeffier M Dechelotte P The role of glutamine in intensive care unit patients: mechanisms of action and clinical outcome Nutr Rev 2005 63 65 69 15762090 10.1111/j.1753-4887.2005.tb00123.x

[B3] Gamrin L Essen P Forsberg AM Hultman E Wernerman J A descriptive study of skeletal muscle metabolism in critically ill patients: free amino acids, energy-rich phosphates, protein, nucleic acids, fat, water, and electrolytes Crit Care Med 1996 24 575 583 10.1097/00003246-199604000-00005 8612406

[B4] Newsholme EA Crabtree B Ardawi MS Glutamine metabolism in lymphocytes: its biochemical, physiological and clinical importance Q J Exp Physiol 1985 70 473 489 3909197 10.1113/expphysiol.1985.sp002935

[B5] Wilmore DW The effect of glutamine supplementation in patients following elective surgery and accidental injury J Nutr 2001 131 2543S 2549S discussion 2550S-2541S 11533310 10.1093/jn/131.9.2543S

[B6] Heyland D Muscedere J Wischmeyer PE Cook D Jones G Albert M Elke G Berger MM Day AG Canadian Critical Care Trials G A randomized trial of glutamine and antioxidants in critically ill patients N Engl J Med 2013 368 1489 1497 10.1056/NEJMoa1212722 23594003

[B7] Oudemans-van Straaten HM Bosman RJ Treskes M van der Spoel HJ Zandstra DF Plasma glutamine depletion and patient outcome in acute ICU admissions Intensive Care Med 2001 27 84 90 10.1007/s001340000703 11280678

[B8] Rodas PC Rooyackers O Hebert C Norberg A Wernerman J Glutamine and glutathione at ICU admission in relation to outcome Clin Sci 2012 122 591 597 10.1042/CS20110520 22248298 PMC3294430

[B9] Dechelotte P Hasselmann M Cynober L Allaouchiche B Coeffier M Hecketsweiler B Merle V Mazerolles M Samba D Guillou YM Petit J Mansoor O Colas G Cohendy R Barnoud D Czernichow P Bleichner G L-alanyl-L-glutamine dipeptide-supplemented total parenteral nutrition reduces infectious complications and glucose intolerance in critically ill patients: the French controlled, randomized, double-blind, multicenter study Crit Care Med 2006 34 598 604 10.1097/01.CCM.0000201004.30750.D1 16505644

[B10] Goeters C Wenn A Mertes N Wempe C Van Aken H Stehle P Bone HG Parenteral L-alanyl-L-glutamine improves 6-month outcome in critically ill patients Crit Care Med 2002 30 2032 2037 10.1097/00003246-200209000-00013 12352037

[B11] Bollhalder L Pfeil AM Tomonaga Y Schwenkglenks M A systematic literature review and meta-analysis of randomized clinical trials of parenteral glutamine supplementation Clin Nutr 2013 32 213 223 10.1016/j.clnu.2012.11.003 23196117

[B12] Lin JJ Chung XJ Yang CY Lau HL A meta-analysis of trials using the intention to treat principle for glutamine supplementation in critically ill patients with burn Burns 2013 39 565 570 10.1016/j.burns.2012.11.008 23313017

[B13] Zheng YM Li F Zhang MM Wu XT Glutamine dipeptide for parenteral nutrition in abdominal surgery: a meta-analysis of randomized controlled trials World J Gastroenterol 2006 12 7537 7541 17167847 10.3748/wjg.v12.i46.7537 PMC4087604

[B14] Zhong Z Liang C Gong S Intravenous glutamine for sever acute pancreatitis: a meta analysis World J Crit Care Med 2013 2 4 8 10.5492/wjccm.v2.i1.4 24701410 PMC3953862

[B15] Duska F Fric M Waldauf P Pazout J Andel M Mokrejs P Tuma P Pachl J Frequent intravenous pulses of growth hormone together with glutamine supplementation in prolonged critical illness after multiple trauma: effects on nitrogen balance, insulin resistance, and substrate oxidation Crit Care Med 2008 36 1707 1713 10.1097/CCM.0b013e318174d499 18496372

[B16] Bellomo R Warrillow SJ Reade MC Why we should be wary of single-center trials Crit Care Med 2009 37 3114 3119 10.1097/CCM.0b013e3181bc7bd5 19789447

[B17] Review Manager (RevMan) [Computer program] 2012 Copenhagen: The Nordic Cochrane Centre, The Cochrane Collaboration

[B18] Higgins JP Thompson SG Quantifying heterogeneity in a meta-analysis Stat Med 2002 21 1539 1558 10.1002/sim.1186 12111919

[B19] Rucker G Schwarzer G Carpenter J Arcsine test for publication bias in meta-analyses with binary outcomes Stat Med 2008 27 746 763 10.1002/sim.2971 17592831

[B20] Andrews PJ Avenell A Noble DW Campbell MK Croal BL Simpson WG Vale LD Battison CG Jenkinson DJ Cook JA Randomised trial of glutamine, selenium, or both, to supplement parenteral nutrition for critically ill patients BMJ 2011 342 d1542 10.1136/bmj.d1542 21415104

[B21] Cai G Yan J Zhang Z Yu Y Immunomodulatory effects of glutamine-enriched nutritional support in elderly patients with severe sepsis: a prospective, randomized, controlled study J Organ Dysfunct 2008 4 31 37 10.1080/17471060701682260

[B22] Cekmen N Aydin A Erdemli O The impact of L-alanyl-L-glutamine dipeptide supplemented total parenteral nutrition on clinical outcome in critically patients E-SPEN, Euro e-J Clin Nutr Metab 2011 6 64 67 10.1016/j.eclnm.2011.02.001

[B23] Eroglu A The effect of intravenous alanyl-glutamine supplementation on plasma glutathione levels in intensive care unit trauma patients receiving enteral nutrition: the results of a randomized controlled trial Anesth Analg 2009 109 502 505 10.1213/ane.0b013e3181a83178 19608826

[B24] Estivariz CF Griffith DP Luo M Szeszycki EE Bazargan N Dave N Daignault NM Bergman GF McNally T Battey CH Furr CE Hao L Ramsay JG Accardi CR Cotsonis GA Jones DP Galloway JR Ziegler TR Efficacy of parenteral nutrition supplemented with glutamine dipeptide to decrease hospital infections in critically ill surgical patients JPEN J Parenter Enteral Nutr 2008 32 389 402 10.1177/0148607108317880 18596310 PMC3062504

[B25] Fuentes-Orozco C Anaya-Prado R Gonzalez-Ojeda A Arenas-Marquez H Cabrera-Pivaral C Cervantes-Guevara G Barrera-Zepeda LM L-alanyl-L-glutamine-supplemented parenteral nutrition improves infectious morbidity in secondary peritonitis Clin Nutr 2004 23 13 21 10.1016/S0261-5614(03)00055-4 14757388

[B26] Fuentes-Orozco C Cervantes-Guevara G Mucino-Hernandez I Lopez-Ortega A Ambriz-Gonzalez G Gutierrez-de-la-Rosa JL Gomez-Herrera E Hermosillo-Sandoval JM Gonzalez-Ojeda A L-alanyl-L-glutamine-supplemented parenteral nutrition decreases infectious morbidity rate in patients with severe acute pancreatitis JPEN J Parenter Enteral Nutr 2008 32 403 411 10.1177/0148607108319797 18596311

[B27] Grau T Bonet A Minambres E Pineiro L Irles JA Robles A Acosta J Herrero I Palacios V Lopez J Blesa A Martinez P The effect of L-alanyl-L-glutamine dipeptide supplemented total parenteral nutrition on infectious morbidity and insulin sensitivity in critically ill patients Crit Care Med 2011 39 1263 1268 10.1097/CCM.0b013e31820eb774 21336131

[B28] Griffiths RD Allen KD Andrews FJ Jones C Infection, multiple organ failure, and survival in the intensive care unit: influence of glutamine-supplemented parenteral nutrition on acquired infection Nutrition 2002 18 546 552 10.1016/S0899-9007(02)00817-1 12093428

[B29] Griffiths RD Jones C Palmer TE Six-month outcome of critically ill patients given glutamine-supplemented parenteral nutrition Nutrition 1997 13 295 302 9178278

[B30] He Xian-li MQJ Lu JG Chu YK Du XL Effect of total parenteral nutrition (TPN) with and without glutamine dipeptide supplementation on outcome in severe acute pancreatitis (SAP) Clin Nutr Suppl 2004 1 43 47 10.1016/j.clnu.2004.07.011

[B31] Luo M Bazargan N Griffith DP Estivariz CF Leader LM Easley KA Daignault NM Hao L Meddings JB Galloway JR Blumberg JB Jones DP Ziegler TR Metabolic effects of enteral versus parenteral alanyl-glutamine dipeptide administration in critically ill patients receiving enteral feeding: a pilot study Clin Nutr 2008 27 297 306 10.1016/j.clnu.2007.12.003 18258342 PMC2692723

[B32] Ozgultekin A Turan G Durmus Y Dincer E Akgun N Comparison of the efficacy of parenteral and branched-chain amino acid solutions given as extra supplements in parallel to the enteral nutrition in head trauma E-SPEN, Euro e-J of Clin Nutr Metab 2008 3 211 216 10.1016/j.eclnm.2008.05.006

[B33] Palmese S Odierna I Scarano D Scibili AC Natale A Pezza M Early enteral nutrition enriched with FOS and intravenous glutamine supplementation in intensive care unit patients Nutr Ther Metab 2006 24 140 146

[B34] Perez-Barcena J Crespi C Regueiro V Marse P Raurich JM Ibanez J Bengoechea JA Garcia De Lorenzo-Mateos A Lack of effect of glutamine administration to boost the innate immune system response in trauma patients in the intensive care unit Crit Care 2010 14 R233 10.1186/cc9388 21184675 PMC3219991

[B35] Perez-Barcena J Regueiro V Marse P Raurich JM Rodriguez A Ibanez J de Lorenzo Mateos AG Bengoechea JA Glutamine as a modulator of the immune system of critical care patients: effect on toll-like receptor expression. A preliminary study Nutrition 2008 24 522 527 10.1016/j.nut.2008.01.056 18367379

[B36] Powell-Tuck J Jamieson CP Bettany GE Obeid O Fawcett HV Archer C Murphy DL A double blind, randomised, controlled trial of glutamine supplementation in parenteral nutrition Gut 1999 45 82 88 10.1136/gut.45.1.82 10369709 PMC1727563

[B37] Sahin H Mercanligil SM Inanc N Ok E Effects of glutamine-enriched total parenteral nutrition on acute pancreatitis Eur J Clin Nutr 2007 61 1429 1434 10.1038/sj.ejcn.1602664 17311061

[B38] Tian H Wang KF Wu TJ Effect of total parenteral nutrition with supplementation of glutamine on the plasma diamine oxidase activity and D-lactate content in patients with multiple organ dysfunction syndrome Zhongguo Wei Zhong Bing Ji Jiu Yi Xue 2006 18 616 618 17038253

[B39] Wernerman J Kirketeig T Andersson B Berthelson H Ersson A Friberg H Guttormsen AB Hendrikx S Pettila V Rossi P Sjoberg F Winso O Scandinavian glutamine trial: a pragmatic multi-centre randomised clinical trial of intensive care unit patients Acta Anaesthesiol Scand 2011 55 812 818 10.1111/j.1399-6576.2011.02453.x 21658010

[B40] Wischmeyer PE Lynch J Liedel J Wolfson R Riehm J Gottlieb L Kahana M Glutamine administration reduces gram-negative bacteremia in severely burned patients: a prospective, randomized, double-blind trial versus isonitrogenous control Crit Care Med 2001 29 2075 2080 10.1097/00003246-200111000-00006 11700398

[B41] Yang SQ Xu JG Effect of glutamine on serum interleukin-8 and tumor necrosis factor-alpha levels in patients with severe pancreatitis Nan Fang Yi Ke Da Xue Xue Bao 2008 28 129 131 18227044

[B42] Ye-Ping Zhou Z-MJ Yong-Hua S Gui-Zhen H Hong S The effects of supplemental glutamine dipeptide on gut integrity and clinical outcome after major escharectomy in severe burns: a randomized, double-blind, controlled clinical trial Clin Nutr Suppl 2004 1 55 60 10.1016/j.clnu.2004.07.012

[B43] Zhang Z Qin HD Ni HB Xu Y Wu HR Cheng H Wang SK Effect of early enriched parenteral alanyl-glutamine on heat shock protein 70 (HSP70) expression in critical patients Zhongguo Wei Zhong Bing Ji Jiu Yi Xue 2007 19 481 484 17708846

[B44] Ziegler T May A Hebbar G Kudsk K Sax H Blumberg H Easley K Wischmeyer P Glutamine dipeptide-supplemented parenteral nutrition in surgical ICU patients: Results of an American randomized, double-blind, multicenter trial Clin Nutr Suppl 2012 7 265

[B45] Novak F Heyland DK Avenell A Drover JW Su X Glutamine supplementation in serious illness: a systematic review of the evidence Crit Care Med 2002 30 2022 2029 10.1097/00003246-200209000-00011 12352035

[B46] Bongers T Griffiths RD McArdle A Exogenous glutamine: the clinical evidence Crit Care Med 2007 35 S545 S552 10.1097/01.CCM.0000279193.23737.06 17713407

[B47] Critical care nutrition website [http://www.criticalcarenutrition.com]

[B48] Vincent JL Preiser JC Are prospective cohort studies an appropriate tool to answer clinical nutrition questions? Curr Opin Clin Nutr Metab Care 2013 16 182 186 10.1097/MCO.0b013e32835d803e 23324900

