# Supplementary material for: Parenteral glutamine supplementation in critical illness: a systematic review
Source: Crit Care. 2014 Apr 18;18(2):R76. doi: 10.1186/cc13836 (PMC4056606; doi:10.1186/cc13836)
Supplement: Additional file 1: Table S1 — Studies of glutamine supplementation in patients not included in the analysis. [file cc13836-S1.docx]

| **Additional file 1: Table S1** | |
| --- | --- |
| **Patients not considered to be critically ill (n =18)** | |
|  | DeBeaux A, O’Riordain M, Ross J, et al. Glutamine supplemented total parenteral nutrition reduces blood mononuclear cell interleukin-8 release in severe acute pancreatitis. Nutrition 1998:14 (3):261-265. |
|  | Morlion BJ, Stehle P, Wachtler P, Siedhoff HP, Köller M, König W, Fürst P, Puchstein C. Total parenteral nutrition with glutamine dipeptide after major abdominal surgery: a randomized, double-blind, controlled study. Ann Surg. 1998 Feb;227(2):302-8. |
|  | Mertes N, Schulzki C, Goeters C, Winde G, Benzing S, Kuhn KS, Van Aken H, Stehle P, Fürst P. Cost containment through L-alanyl-L-glutamine supplemented total parenteral nutrition after major abdominal surgery: a prospective randomized double-blind controlled study. Clin Nutr. 2000 Dec;19(6):395-401. |
|  | Ockenga J, Borchert K, Rifai K, Manns MP, Bischoff SC. Effect of glutamine-enriched total parenteral nutrition in patients with acute pancreatitis. Clin Nutr 2002;21(5):409-16. |
|  | Fläring UB, Rooyackers OE, Wernerman J, Hammarqvist F. Glutamine attenuates post-traumatic glutathione depletion in human muscle. Clin Sci (Lond). 2003 Mar;104(3):275-82. |
|  | Jiang Z, Jiang H, Furst P. The impact of glutamine dipeptides on outcome of surgical patients: systematic review of randomized controlled trials from Europe and Asia. Clinical Nutrition Supplements 2004;1(1):17-23. |
|  | Jing-Xiang S, Xiao-Huang T, Lie W, Chen-Jin L. Glutamine dipeptide-supplemented parenteral nutrition in patients with colorectal cancer. Clinical Nutrition Supplements 2004, 1(1):49-53. |
|  | Blijlevens NM, Donnelly JP, Naber AH, Schattenberg AV, DePauw BE. A randomised, double-blinded, placebo-controlled, pilot study of parenteral glutamine for allogeneic stem cell transplant patients. Support Care Cancer. 2005 Oct;13(10):790-6. Epub 2005 Mar 15. |
|  | Lin MT, Kung SP, Yeh SL, Liaw KY, Wang MY, Kuo ML, Lee PH, Chen WJ. Glutamine-supplemented total parenteral nutrition attenuates plasma interleukin-6 in surgical patients with lower disease severity. World J Gastroenterol. 2005 Oct 21;11(39):6197-201. |
|  | Ockenga J, Borchert K, Stüber E, Lochs H, Manns MP, Bischoff SC. Glutamine-enriched total parenteral nutrition in patients with inflammatory bowel disease. Eur J Clin Nutr. 2005 Nov;59(11):1302-9. |
|  | Yao GX, Xue XB, Jiang ZM, Yang NF, Wilmore DW. Effects of perioperative parenteral glutamine-dipeptide supplementation on plasma endotoxin level, plasma endotoxin inactivation capacity and clinical outcome. Clin Nutr. 2005 Aug;24(4):510-5. |
|  | Kumar S, Kumar R, Sharma SB, Jain BK. Effect of oral glutamine administration on oxidative stress, morbidity and mortality in critically ill surgical patients. Indian J Gastroenterol. 2007 Mar-Apr;26(2):70-3. |
|  | Sornsuvit C, Komindr S, Chuncharunee S, Wanikiat P, Archararit N, Santanirand P. Pilot Study: effects of parenteral glutamine dipeptide supplementation on neutrophil functions and prevention of chemotherapy-induced side-effects in acute myeloid leukaemia patients. J Int Med Res. 2008 Nov-Dec;36(6):1383-91. PubMed PMID: 19094450. |
|  | Yeh CN, Lee HL, Liu YY, Chiang KC, Hwang TL, Jan YY, Chen MF. The role of parenteral glutamine supplement for surgical patient perioperatively: result of a single center, prospective and controlled study. Langenbecks Arch Surg. 2008 Nov;393(6):849-55. Epub 2008 Aug 20. |
|  | Asprer JM, Llido LO, Sinamban R, Schlotzer E, Kulkarni H. Effect on immune indices of preoperative intravenous glutamine dipeptide supplementation in malnourished abdominal surgery patients in the preoperative and postoperative periods. Nutrition. 2009 Sep;25(9):920-5. |
|  | Fan YP, Yu JC, Kang WM, Zhang Q. Effects of glutamine supplementation on patients undergoing abdominal surgery. Chin Med Sci J. 2009 Mar;24(1):55-9. |
|  | Lu CY, Shih YL, Sun LC, Chuang JF, Ma CJ, Chen FM, Wu DC, Hsieh JS, Wang JY. The inflammatory modulation effect of glutamine-enriched total parenteral nutrition in postoperative gastrointestinal cancer patients. Am Surg. 2011 Jan;77(1):59-64. PubMed PMID: 21396307. |
|  | Hajdu N, Belagyi T, Issekutz A, Bartek P, Gartner B, Olah A. [Intravenous glutamine and early nasojejunal nutrition in severe acute pancreatitis - a prospective randomized clinical study]. Magyar sebeszet 2012;65(2):44-51. |
| **No clinical outcomes (n =5)** | |
|  | Umpleby AM, Carroll PV, Russell-Jones DL, Treacher DF, Jackson NC. Glutamine supplementation and GH/IGF-I treatment in critically ill patients: effects on glutamine metabolism and protein balance. Nutrition 2002;18(2):127-9. |
|  | Berg A, Rooyackers O, Norberg A, Wernerman J. Elimination kinetics of L-alanyl-L-glutamine in ICU patients. Amino Acids. 2005 Nov;29(3):221-8. Epub 2005 Aug 1. |
|  | Cetinbas F, Yelken B, Gulbas Z. Role of glutamine administration on cellular immunity after total parenteral nutrition enriched with glutamine in patients with systemic inflammatory response syndrome. J Crit Care. 2010 Dec;25(4):661.e1-6. PubMed PMID: 20537501 |
|  | Mondello S, Italiano D, Giacobbe MS, Mondello P, Trimarchi G, Aloisi C, Bramanti P, Spina E. Glutamine-supplemented total parenteral nutrition improves immunological status in anorectic patients. Nutrition. 2010 Jun;26(6):677-81. Epub 2010 Feb 1. PubMed PMID: 20122815. |
|  | Bakalar B, Duska F, Pachl J, Fric M, Otahal M, Pazout J, Andel M. Parenterally administered dipeptide alanyl-glutamine prevents worsening of insulin sensitivity in multiple-trauma patients. Crit Care Med. 2006 Feb;34(2):381-6. |
| **Were duplicates or subgroups of included studies (n =4)** | |
|  | Griffiths RD, Allen KD, Andrews FJ, Jones C. Infection, multiple organ failure, and survival in the intensive care unit: influence of glutamine-supplemented parenteral nutrition on acquired infection. Nutrition 2002;18(7-8):546-52. |
|  | Ziegler TR, Ogden LG, Singleton KD et al. Parenteral glutamine increases serum heat shock protein 70 in critically ill patients. Intensive Care Med 2005;31(8):1079-86. |
|  | Luo M, Fernandez-Estivariz C, Jones DP, Accardi CR, Alteheld B, Bazargan N, Hao L, Griffith DP, Blumberg JB, Galloway JR, Ziegler TR. Depletion of plasma antioxidants in surgical intensive care unit patients requiring parenteral feeding: effects of parenteral nutrition with or without alanyl-glutamine dipeptide supplementation. Nutrition. 2008 Jan;24(1):37-44. PubMed PMID: 18065204; PubMed Central PMCID: PMC2712494. |
| **Crossover design studies (n =2)** | |
|  | Berg A, Bellander BM, Wanecek M, Norberg A, Ungerstedt U, Rooyackers O, Wernerman J. The pattern of amino acid exchange across the brain is unaffected by intravenous glutamine supplementation in head trauma patients. Clin Nutr. 2008 Dec;27(6):816-21. Epub 2008 Jul 22. |
|  | Berg A, Bellander BM, Wanecek M, Gamrin L, Elving A, Rooyackers O, Ungerstedt U, Wernerman J. Intensive Intravenous glutamine supplementation to head trauma patients leaves cerebral glutamate concentration unaffected. Int Care Med. 2006 Nov;32(11):1741-6. Epub 2006 Sep 23. |
| **Varying doses of glutamine (n =1)** | |
|  | Tjader I, Rooyackers O, Forsberg AM, Vesali RF, Garlick PJ, Wernerman J. Intensive Care Med 2004;30(2):266-75. |
| **Combined enteral and parenteral glutamine (n =1)** | |
|  | Heyland D, Muscedere J, Wischmeyer PE, Cook D, Jones G, Albert M, Elke G, Berger MM, Day AG for the Canadian Critical Care Trials Group. A Randomized Trial of Glutamine and Antioxidants in Critically Ill Patients. N Engl J Med 2013;368(16):1487-95. |
| **Questionably low dosage of glutamine(0.002 gm/kg/day) (n =1)** | |
|  | Yang D, Xu J. Effect of dipeptide of glutamine and alanine on severe traumatic brain injury. Chin J Traumatology 2007;10(3):145-149. |
| **Reported data from a subgroup of its study population (n -1)** | |
|  | Goeters C, Wenn A, Mertes N, Wempe C, Van Aken H, Stehle P, Bone HG. Parenteral L-alanyl-L-glutamine improves 6-month outcome in critically ill patients. Crit Care Med. 2002 Sep; 30(9): 2032-7. |
